# Euthanasia - A Novel Intricacy for Psychiatry’s Purview?

**DOI:** 10.1192/j.eurpsy.2024.687

**Published:** 2024-08-27

**Authors:** Í. Alberdi-Páramo, M. Pérez-Lombardo, J. Pemán-Rodríguez, J. E. Ibáñez Vizoso, R. Á. Baena Mures, L. Niell Galmés, J. Rodríguez Quijano, G. Montero-Hernández

**Affiliations:** ^1^Psiquiatría, Hospital Clínico San Carlos; ^2^Universidad Complutense de Madrid, Madrid; ^3^Psiquiatría, Hospital Nuestra Señora Candelaria, Tenerife; ^4^Psiquiatría, Fundación Alcorcón, Madrid; ^5^Psiquiatría, Red Salud Mental Vizkaya, Bilbao, Spain

## Abstract

**Introduction:**

Numerous countries, notably within Europe, have sanctioned the practice of euthanasia. Extant legal frameworks meticulously define the extent, essence, and application of euthanasia, encompassing divergent characterizations, explications of entitlements, procedural modalities, and provisions for access. Nonetheless, the precise function of psychiatrists within these legislative contours remains conspicuously nebulous.

**Objectives:**

The present inquiry undertakes a comprehensive evaluative review of the euthanasia phenomenon vis-à-vis the intricate tapestry of European legislative paradigms, with an emphasis on elucidating the multifaceted involvement of psychiatry within this evolving landscape.

**Methods:**

A nuanced narrative review is undertaken, encapsulating the contemporary state-of-affairs, fundamental conceptual architectures, the tenets of the Spanish Organic Law 03/2021, and the pharmaceutic armamentarium deployed in the orchestration of euthanasic practices. Additionally, the methodological blueprint employed within a prominent tertiary healthcare institution situated in Madrid is meticulously expounded.

**Results:**

To date, euthanasia has garnered legal imprimatur across diverse jurisdictions including, but not limited to, the Netherlands, Belgium, Colombia, Canada, Spain, and New Zealand. The ambit of assisted death and its application to the domain of mental infirmities is meticulously deconstructed. Within the overarching realm of foundational concepts, a rigorous delineation is rendered between euthanasia, medical succor in the throes of mortality, assisted self-termination, facilitated demise, provision of mortal release, judicious calibration of therapeutic enterprise, and the contours of palliative sedation. Distinction between the principal executor and the advisory consultant is rendered salient. The rubric of conscientious objection emerges as an inviolable entitlement of healthcare practitioners enmeshed in the provisionary matrix.

The enduring incumbency of the psychiatrist as a pivotal appraiser of cognitive and volitional faculties holds firm. The conspicuous influence of psychopathological constellations upon the contours of euthanasia eligibility precipitates cogent deliberation.

**Conclusions:**

As the frontiers of euthanasia expand to encompass an augmented array of legal jurisdictions, this study underscores the increasingly intricate role inhabited by psychiatrists in the matrix of evaluative assessments. The proclivity of mental maladies to exert a substantial gravitational pull upon determinations of eligibility for euthanasia accentuates the exigency for refined explication of roles and responsibilities within this evolving sphere, a clarion call resonant not only within the precincts of psychiatry but reverberating across the broader firmament of medical praxis.

**Disclosure of Interest:**

None Declared

